# Tracheal Lobular Capillary Haemangioma: A Rare Benign Cause of Recurrent Haemoptysis

**DOI:** 10.1155/2016/6290424

**Published:** 2016-09-25

**Authors:** Metesh Nalin Acharya, Konstantinos Kotidis, Mahmoud Loubani

**Affiliations:** Department of Cardiothoracic Surgery, Castle Hill Hospital, Cottingham, UK

## Abstract

Lobular capillary haemangioma (LCH), previously known as pyogenic granuloma, is a benign vascular lesion commonly found within the oral and nasal cavity. However, it is rarely encountered within the trachea, where presenting features include recurrent haemoptysis, cough, and wheeze. We here describe a case of a 7 mm tracheal LCH in a 56-year-old woman, which was successfully resected at interventional bronchoscopy using biopsy forceps. Clinicians should be aware of tracheal LCH in the differential diagnosis for recurrent haemoptysis.

## 1. Introduction

Tracheal tumours represent only 2% of all upper respiratory tract tumours [1]. Those in adults are usually malignant; benign tumours, including chondroma, papilloma, and fibroma most often occur in the proximal third of the trachea in adults and its distal third in children [2]. Lobular capillary haemangioma (LCH), previously called pyogenic granuloma, commonly presents on the lip, nose, oral cavity, or tongue [3, 4] but has rarely been reported within the trachea. We here report the case of a 56-year-old woman with a tracheal LCH managed by bronchoscopic resection using biopsy forceps. 

## 2. Case Presentation

A 56-year-old Caucasian woman was referred to our institution with several episodes of mild haemoptysis over the preceding three months. She denied associated chest pain, exertional dyspnoea, dysphagia, syncope, weight loss, anorexia, fever, and rigors. There was no history of airway instrumentation or foreign body aspiration. Her medical background included laparoscopic sterilisation, oesophagitis, meningitis, and hypertension. She was a nonsmoker and consumed alcohol within normal limits.

**Figure 1:**
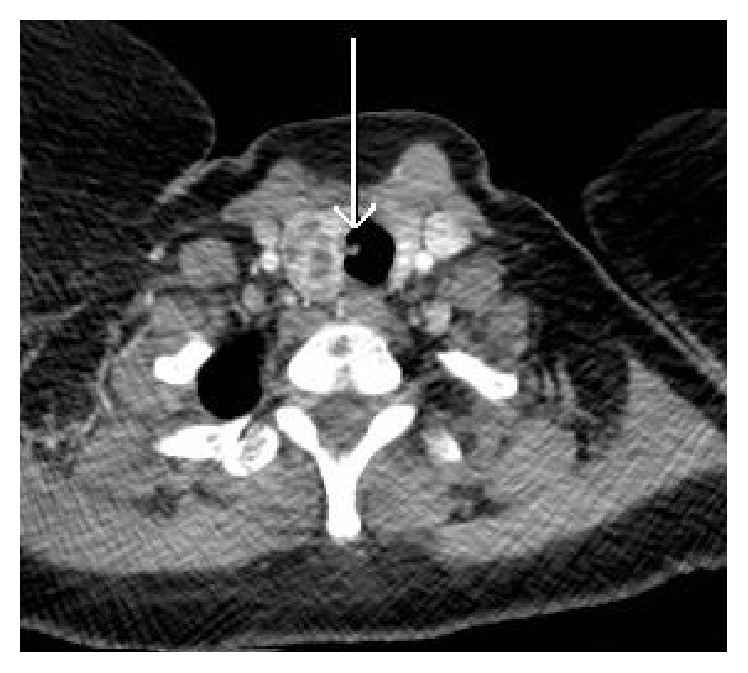
Computed tomography scan demonstrating a pedunculated lesion (arrow) projecting from the right tracheal wall into its lumen.

Physical examination was unremarkable and routine blood investigations and chest radiograph were normal. Pulmonary function was satisfactory with FEV_1_ 3.50 L and FVC 3.85 L. Computed tomography (CT) scanning (Figure 1) demonstrated a pedunculated lesion arising from the right tracheal wall and projecting into its lumen. Following case discussion at a multidisciplinary team meeting, patient consent was obtained for rigid bronchoscopy under general anaesthetic. A 7 mm polypoid mass with a small pedicle was identified 2 cm inferior to the vocal cords on the right tracheal wall and resected in entirety utilising biopsy forceps. Haemostasis was achieved following local application of electrocautery and adrenaline-soaked swabs, and the patient was discharged home the next day. The patient was asymptomatic at six-month and one-year follow-up with no radiological or bronchoscopic evidence of disease recurrence.

**Figure 2:**
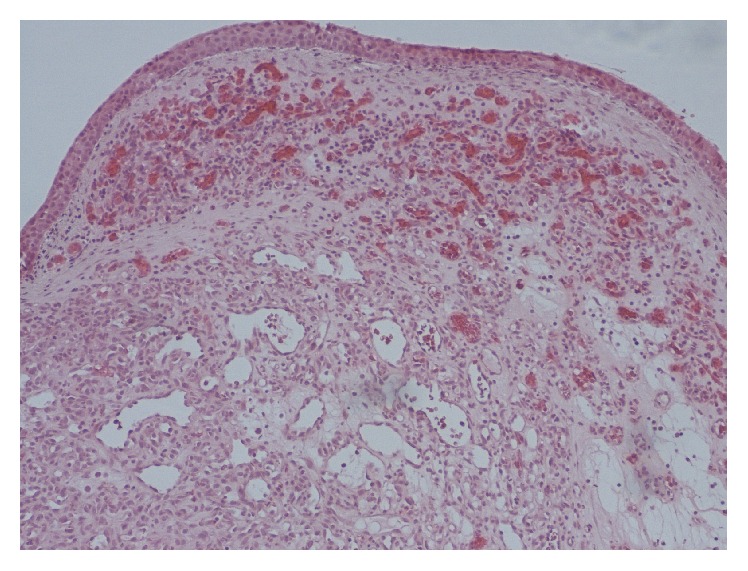
Histopathological analysis of the resected specimen demonstrates capillary haemangioma covered by metaplastic squamous epithelium (haematoxylin and eosin stain; original magnification ×100).

Histological examination (Figure 2) of the surgical specimen demonstrated nodular proliferation of endothelial cells and capillary-type lumina separated by mildly oedematous and inflamed stroma, suggestive of a capillary haemangioma. There was no pathological evidence of dysplasia or malignancy.

## 3. Discussion

Tracheal tumours account for less than 2% of all upper respiratory tract tumours [1] and are usually malignant in adult populations. Commoner benign tumours in this region include chondroma, papilloma, and fibroma, with less than 10% being vascular in origin [5].

Lobular capillary haemangioma is a benign lesion characterised microscopically by a distinctive lobular arrangement of capillaries in an oedematous, fibroblastic stoma [2, 3, 6]. The traditionally ascribed term pyogenic granuloma is inaccurate, since the tumour neither is caused by bacterial infection, nor is a true granuloma [7]. Younger lesions are typically vascular in appearance but may become collagenous with age [8]. Tracheal LCH usually presents as a painless red-purple nodule or papule associated with cough, wheeze, or haemoptysis, developing rapidly over a period of days to weeks. It may rarely manifest with airway haemorrhage [9] or obstruction [10].

The surface epithelium is often ulcerated, predisposing to invasion by microorganisms. Commonly found on the lip, nose, oral cavity, and tongue [3, 4], occurrence within the tracheobronchial tree is extremely rare and infrequently described in medical literature.

Although the precise aetiological mechanisms accounting for LCH are not yet fully ascertained, traumatic insults, hormonal imbalances during pregnancy, viral and bacterial infections, microscopic arteriovenous malformations, angiogenic growth factors, and cytogenetic abnormalities have been proposed as causative factors.

Chest radiography and computed tomography findings may be inconclusive, and thus bronchoscopy with biopsy plays a key role in diagnosis of tracheal LCH, whilst additionally affording the opportunity for therapeutic intervention. Endoscopic appearances are nonspecific and may mimic adenoma, carcinoma, or carcinoid tumour. For this reason, decision for surgical resection should be avoided until a definitive histopathological diagnosis is established.

The extent and size of the lesion, as well as patient age and comorbidities, require consideration prior to any therapeutic intervention for LCH. Despite their benign nature, local recurrence is common and thus surgical excision remains the treatment of choice. Nevertheless, mucocutaneous LCH is also amenable to various nonsurgical techniques including snare electrocautery, cryotherapy, YAG laser therapy, and plaque radiation. In the present case, we were able to safely remove the culprit lesion with biopsy forceps alone and with minimal bleeding owing to its small vascular pedicle.

To our knowledge, there are only 14 reports of tracheal LCH in English medical literature to date (Table 1). Irani et al. successfully extracted a 2-3 mm LCH occurring 3 cm below the vocal cords in a 72-year-old female with flexible biopsy forceps [3]. Endoscopic techniques were employed by Xu et al. [11] and Madhumita et al. [2], who resected a 4 mm tracheal LCH in a 64-year-old male and a 1 cm tracheal LCH in a 40-year-old female, respectively. Chawla et al. combined endoscopic excision and laser therapy for distal tracheal LCH in a 62-year-old male [12]. Chen et al. utilised cryotherapy to remove a 2 cm tracheal LCH which occluded the majority of the tracheal lumen in a 14-year-old girl [13]. Cryotherapy was similarly applied for a 1.5 cm tracheal LCH near the carina in the report by Udoji and Bechara [14]. Two 4 mm lesions were excised with biopsy forceps and electrocautery in Porfyridis's report of a 17-year-old boy with recurrent haemoptysis [6]. Electrocautery loop snaring was also used in a 22-year-old male with a 1.5 cm LCH of the posterior tracheal wall in the report by Amy and Enrique [4], as well as in a 57-year-old male by Kalanjeri et al. [15]. Following multiple poor responses to electrocautery and argon plasma coagulation, Shen et al. [16] utilised brachytherapy successfully to control a 2 cm tracheal LCH in a 35-year-old male. Dabó et al. required photocoagulation to achieve haemostasis following significant bleeding on removal of a tracheal LCH using rigid biopsy forceps in a 51-year-old female [17]. Zambudio et al. performed arterial embolisation to control massive haemoptysis from a bleeding tracheal capillary haemangioma in a 66-year-old female with thrombocytopaenia [9]. Circulatory assistance with extracorporeal membrane oxygenation was employed as a precautionary measure when debulking a large LCH in a 23-year-old pregnant female in the report by Prakash et al. [10]. Putora et al. propose this lesion occurring as a consequence of erlotinib chemotherapy in a 64-year-old patient with squamous cell lung cancer; interestingly, complete resolution of the LCH was noted on discontinuation of erlotinib and no invasive intervention was necessary [18].

In conclusion, LCH is a benign lesion rarely found within the trachea. Common presenting features may include recurrent haemoptysis, cough, and wheeze. Symptomatic lesions are usually amenable to direct evaluation and removal via interventional bronchoscopic techniques.

## Figures and Tables

**Table 1:** Summary of previously reported cases of tracheal lobular capillary haemangioma.

Author	Age (years), M/F	Tumour size	Tumour location	Treatment	Outcome
Madhumita et al. [2]	40, F	10 × 5 mm	Upper third of right anterolateral tracheal wall	Endoscopic resection	Good at 1 year

Irani et al. [3]	72, F	2-3 mm	3 cm below vocal cords	Endoscopic resection	Good at 1 year

Amy and Enrique [4]	22, M	10–15 mm	3 cm above carina on left posterior tracheal wall	Electrocautery	Good

Porfyridis et al. [6]	17, M	4 mm	Upper third of left anterolateral tracheal wall	Endoscopic resection	Good at 1 year

Zambudio et al. [9]	66, F	Occluding 30–40% of airway	Between first and third tracheal rings	Embolisation	Good at 1 year

Prakash et al. [10]	23, F	20 × 40 mm	Posterior tracheal wall	Endoscopic resection with extracorporeal membrane oxygenation	Good

Xu et al. [11]	64, M	3-4 mm	Left anterolateral tracheal wall	Endoscopic resection	Good at 8 months

Chawla et al. [12]	62, M	Unknown	Distal right tracheal wall	Endoscopic resection and laser therapy	Unknown

Chen et al. [13]	14, F	15–20 mm	Lower third of anterior tracheal wall	Cryotherapy and argon plasma coagulation	Good at 3 months

Udoji and Bechara [14]	55, M	4 × 5 mm	Distal left lateral tracheal wall	Cryotherapy	Good at 3 months

Kalanjeri et al. [15]	57, M	Occluding 70% of airway	Posterior middle tracheal wall	Electrocautery	Unknown

Shen et al. [16]	35, M	15–20 mm	Lateral wall of proximal left main bronchus	Brachytherapy	Good at 2 years

Dabó et al. [17]	51, F	Unknown	Lower third of left lateral tracheal wall	Endoscopic resection and laser photocoagulation	Good at 27 months

Putora et al. [18]	64, M	Unknown	Distal tracheal wall	Spontaneous remission on cessation of erlotinib for lung cancer	Good

Present case	56, F	7 mm	2 cm below vocal cords on right tracheal wall	Endoscopic resection and electrocautery	Good at 1 year
